# Efficient conformational space exploration in *ab initio* protein folding simulation

**DOI:** 10.1098/rsos.150238

**Published:** 2015-08-26

**Authors:** Ahammed Ullah, Nasif Ahmed, Subrata Dey Pappu, Swakkhar Shatabda, A. Z. M. Dayem Ullah, M. Sohel Rahman

**Affiliations:** 1 AℓEDA Group, Department of CSE, BUET, ECE Building, Dhaka 1205, Bangladesh; 2 Department of CSE, Independent University, Bangladesh, Dhaka 1229, Bangladesh; 3 Department of CSE, United International University, Dhanmondi, Dhaka 1209, Bangladesh; 4 Barts Cancer Institute, Queen Mary University of London, London, UK

**Keywords:** protein folding simulation, protein structure prediction, energy function, optimization, discrete lattices, genetic algorithms

## Abstract

*Ab initio* protein folding simulation largely depends on knowledge-based energy functions that are derived from known protein structures using statistical methods. These knowledge-based energy functions provide us with a good approximation of real protein energetics. However, these energy functions are not very informative for search algorithms and fail to distinguish the types of amino acid interactions that contribute largely to the energy function from those that do not. As a result, search algorithms frequently get trapped into the local minima. On the other hand, the hydrophobic–polar (HP) model considers hydrophobic interactions only. The simplified nature of HP energy function makes it limited only to a low-resolution model. In this paper, we present a strategy to derive a non-uniform scaled version of the real 20×20 pairwise energy function. The non-uniform scaling helps tackle the difficulty faced by a real energy function, whereas the integration of 20×20 pairwise information overcomes the limitations faced by the HP energy function. Here, we have applied a derived energy function with a genetic algorithm on discrete lattices. On a standard set of benchmark protein sequences, our approach significantly outperforms the state-of-the-art methods for similar models. Our approach has been able to explore regions of the conformational space which all the previous methods have failed to explore. Effectiveness of the derived energy function is presented by showing qualitative differences and similarities of the sampled structures to the native structures. Number of objective function evaluation in a single run of the algorithm is used as a comparison metric to demonstrate efficiency.

## 1. Introduction

Proteins coordinate most of the cellular functions in an organism. The tertiary structure of a protein largely determines its functionality. An irregularly folded protein loses its functionality and is the cause of many diseases. The knowledge of the folding process and the native structure of proteins can help us to distinguish between a properly folded protein and a mis-folded protein. Hence, protein folding simulation or protein structure prediction is crucial in medicine for designing new drugs as well as in biotechnology for designing novel enzymes. Given the primary structure of a protein, the problem of protein structure prediction is to find its three-dimensional native structure. It has been one of the most challenging problems in molecular biology for several decades. The primary structure of a protein is composed of a linear sequence of amino acid residues. The tertiary or three-dimensional structure in the native state is postulated to have the minimum free energy. This postulation is due to the Nobel laureate Christian B. Anfinsen's famous work on the relationship between the amino acid sequence and the biologically active conformation [1]. Since Anfinsen's thermodynamic hypothesis, it has been believed that the native structure of a protein is a unique, stable and kinetically accessible structure with minimum free energy and it is encoded in its primary amino acid sequence. However, because of the complex nature of the folding process and the unknown contributing factors of the energy function, how and why a protein adopts its specific structure remains as one of the top outstanding issues in modern science.

Worldwide genome projects for large-scale DNA sequencing have determined massive amounts of primary structure data. In structural genomics, community-wide efforts are invested to experimentally determine proteins' tertiary structures using expensive and time consuming X-ray crystallography or NMR spectroscopy. Despite the efforts, the number of primary structures mapped to their corresponding tertiary structures is very small compared with the total number of sequenced proteins. For the successful prediction of tertiary structures of the exponentially increasing primary sequences of proteins, there is now more than ever an emphasis on the computational approach, especially with the advent of modern high computing power. In 2013, Karplus, Levitt and Warshel received the Nobel Prize in Chemistry for their pioneering work in protein folding simulations using computers. However, the primary structure of a protein has a very large number of degrees of freedom. Thus, it is possible for it to fold into an astronomical number of structures. The time required for a protein to fold into its native state by searching all the possible structures seems infeasible. But natural protein folding is a rapid, spontaneous process. This dual property of proteins is known as the Levinthal paradox [2]. This paradox rules out the possibility of protein folding being a random process and has inspired development of well-guided computational approaches for protein structure prediction.

The computational protein structure prediction approaches can be broadly classified into three categories: homology modelling, threading or fold recognition and *ab initio* methods. The first two categories of approaches prune the astronomically large search space of possible protein structures assuming that the protein pursued adopts a structure that is close to the experimentally determined structure of another protein. However, homology modelling and fold recognition approaches do not help to answer the question of how and why a protein adopts its specific structure. Moreover, if a homologous structure does not exist or cannot be identified, the native structure has to be predicted from scratch, i.e. by an *ab initio* approach. The *ab initio* approach depends solely on the primary sequence of a protein and is the only approach for finding the pathway of the protein folding process. The major bottleneck of an *ab initio* approach is that it requires vast computational resources depending on the details of the model and the representation of the structure. Owing to the current computational capacity constraint, relatively small proteins have been considered in this approach so far. More computational resources and better algorithms are needed to predict large protein structures in this approach. A comparative study on various aspects of *ab initio* approaches can be found in Lee *et al.* [3].

The use of optimization methods to search for the minimum energy state of a protein dates back to 1953 when Corey & Pauling [4] proposed one of the first energy functions for the rotation of peptide bonds. A large number of physics-based or knowledge-based statistical energy functions [5,6] have been used in protein structure prediction along with different methods and algorithms. Typically, a search algorithm in the *ab initio* approach for protein structure prediction explores a search space of feasible structure decoys also called conformations. A conformation is a placement of the amino acids or necessary atoms in the three-dimensional space. Neighbouring pairs of amino acids in a conformation are said to form contacts. Energy functions usually assign energy potential for different types of contacts formed in a conformation. In the low-resolution hydrophobic–polar (HP) energy model [7], 20 different amino acids are divided into two categories: hydrophobic (H) and polar (P). The HP model assigns unit negative energy only for H–H interactions. HP energy function is only useful to drive the search initially but is not very helpful for the later stages. Additionally, it is not able to distinguish the native state due to the large number of degenerate states. More elaborate energy functions that consider 20×20 pairwise amino acid interactions are derived by applying statistical methods over X-ray crystallography and NMR data [8,9]. These energy functions are much more informative for the later stages of the search but are less informative for guiding the search to avoid local minima. Current state-of-the-art results in *ab initio* protein structure prediction have been achieved by using HP energy function initially as well as in the intermediate stages of the search and by resorting to real energy functions in the rest of the stages [10–13].

Theoretically the search space is conjectured to be funnel shaped but the energy landscape provided by the elaborated energy functions is very rugged shaped. The real energy function does not contain any information for the search algorithm to distinguish a large magnitude of contact energy from an accumulation of small magnitude thereof. As a result, the search traps into local minima frequently. If a contact having large magnitude of energy can be formed initially by sacrificing some contacts having small magnitude of energy then this would increase the degree of freedom for the later search stages when it is possible to try to re-establish the sacrificed contacts.

In this paper, we present a strategy to derive a more informed energy function for the search algorithm by applying a non-uniform scaling on the real energy function. Our derived energy function encapsulates the idea of increasing the separation between the large and small magnitude of energy in a way so as to aid a search algorithm in effectively distinguishing among the contacts with different energy potentials. The idea also includes the trade-off to be made when separations become too greedy. The greediness occurs when very large separation is used at which point the algorithm will favour only a small number of contacts. With the separation achieved by a non-uniform scaling our derived energy function helps the search algorithm to avoid local minima. Integration of 20×20 pairwise information makes our derived energy function free from the constraints and limitations of the simplified HP energy function. The effectiveness of this derived energy function is verified qualitatively by observing the differences of energy distributions produced by different energy functions as well as empirically from the analysis of the experimental data. We also show that our derived energy function helps the search to converge in less time as compared with the previous approaches. A brief summary of the related work in the literature is presented in the rest of the section.

### 1.1 Related work

The protein folding simulation or protein structure prediction problem is a hard combinatorial problem even in the simple square [14] and cubic lattices [15]. Applying exhaustive search techniques is not suitable for this problem due to the astronomically large search space. Several algorithmic frameworks have been extensively used in the literature to solve this problem including approximation algorithms, local search, constraint programming, population-based local search, genetic algorithm, particle swarm optimization, etc. Large number of works are found in the literature in the earlier stages on deterministic and approximation algorithms [16]. Local search methods, albeit incomplete, provide good solutions quickly. Previous approaches using local search methods for protein structure prediction include tabu search [17,18], simulated annealing [19] and population-based local search methods [20]. Shatabda *et al.* [13] presented a mixed heuristic local search algorithm. The mixed heuristic local search evaluates the generated neighbouring solutions of the current solution by randomly selecting a heuristic from a given number of heuristics designed by the authors.

Dal Palu *et al.* [21] applied heuristic approaches to protein folding problem using constraint logic programming over finite domains (CLP(FD)). Despite a satisfactory performance on small-/medium-sized instances, it was proved ineffective in scaling to larger instances of the problem [22]. Later, they developed COLA solver that can model the protein folding as a constraint satisfaction problem (CSP) on three-dimensional lattices and produce acceptable quality solutions for larger proteins [23]. Ullah *et al.* [24] proposed a two-stage optimization approach combining constraint programming and local search. The first stage of this approach produces compact optimal structures by using the CPSP tools [25] based on the HP model. In the second stage, those compact structures are used as the input of a simulated annealing-based local search that is guided by the energy model of Berrera *et al.* [8] (BM energy model). In another hybrid approach, Ullah & Steinhofel [11] applied a constraint programming-based large neighbourhood search technique on top of the of COLA solver [23]. This hybrid approach produced the state-of-the-art results for several small sized benchmark proteins.

In particle swarm optimization category, a recent work of Maher *et al.* [26] uses a firefly inspired method. Their work employs a more effective metric for comparison, namely the number of energy function evaluation in a single run of the algorithm. A recent work of Rashid *et al.* [12] on a population-based genetic algorithm used a mixed energy model during the entire lifetime of the search. Their mixed energy model used both HP and 20-pairwise BM energy functions in a single algorithm and were able to produce state-of-the-art results within a pre-specified cut-off time. So far, most of the works in the literature have experimented on small-sized proteins, i.e. having less than 75 amino acid residues. To the best of our knowledge, Shatabda *et al.* [13] first worked on proteins having up to 160 amino acid residues. Later, their work was outperformed by Rashid *et al.* [12] in all the benchmark proteins having lengths from 54 to 160.

The rest of the paper is organized as follows: §2 provides the description of materials and methods, experimental results and discussion are presented in §3 and we conclude briefly in §4 with a summary of the work and possible future directions.

## 2. Material and methods

The computational complexity of searching for a native structure depends on the underlying model to represent the protein structure. Models with all-atomic details and off-lattice protein modelling pose much complexity on the representation and require huge computational time [27]. For this reason, reduced models have been considered by many researchers. In the initial phase of hierarchical protein structure prediction, a general practice is to generate a large number of sample decoys or candidate structures using a reduced model. Among them only several thousands are selected for the next stage. These selected decoys are then further refined by adding necessary backbone and side-chain atoms [28,29]. Often the search methods in the reduced models are guided by contact-based energy functions and the conformation, i.e. the amino acid positions are restricted to discretized lattices. These are successfully used in investigating the protein folding process in detail [30,31]. Discretized lattice representations have also been used in CASP competition by one of the best performing systems, namely TASSER [32].

### 2.1 Discretized lattice model

On-lattice protein structure prediction can produce very good approximation of real protein structures if the underlying lattice model is chosen carefully. Different discretized lattice models have been considered in the literature. Among those, the face centered cubic (FCC) lattice has been shown to yield very good approximations of real protein structures [33,34]. The domain of an FCC lattice consists of points (x,y,z) ∈ Z. Two FCC points *p*_*i*_≡(*x*_*i*_,*y*_*i*_,*z*_*i*_) and *p*_*j*_≡(*x*_*j*_,*y*_*j*_,*z*_*j*_) are adjacent if |*x*_*i*_−*x*_*j*_|≤1, |*y*_*i*_−*y*_*j*_|≤1, |*z*_*i*_−*z*_*j*_|≤1 and |*x*_*i*_−*x*_*j*_|+|*y*_*i*_−*y*_*j*_|+|*z*_*i*_−*z*_*j*_|=2. The primary structure of a protein comprises a sequence of *n* amino acids, i.e. **s**=*s*_1_,…,*s*_*n*_ where each $s_{i}\in A$. Here, A is the alphabet of amino acids and |A|=20. Each amino acid monomer is represented by a single point in the three-dimensional space. This point is mapped to a valid point in the FCC lattice. This constraint is also called the *lattice constraint*. Every pair of consecutive amino acids in the sequence must also be adjacent or neighbours in the lattice. This is also called the *chain constraint* which ensures the connectivity of the consecutive amino acid monomers. Moreover, two monomers cannot occupy the same point in the lattice in order to avoid any steric clashes. This last constraint is often called the *self-avoiding walk constraint*.

Two amino acids *s*_*a*_ and *s*_*b*_ mapped to the lattice points *p*_*i*_≡(*x*_*i*_,*y*_*i*_,*z*_*i*_) and *p*_*j*_≡(*x*_*j*_,*y*_*j*_,*z*_*j*_), respectively, are said to be in contact if and only if they are not adjacent in the primary sequence *s*, i.e. |*a*−*b*|>1 and the points they are mapped to are adjacent in the FCC lattice, i.e. |*x*_*i*_−*x*_*j*_|+|*y*_*i*_−*y*_*j*_|+|*z*_*i*_−*z*_*j*_|=2. More formally, contact(*i*,*j*)=1, if |*x*_*i*_−*x*_*j*_|+|*y*_*i*_−*y*_*j*_|+|*z*_*i*_−*z*_*j*_|=2 and |*a*−*b*|>1; and contact(*i*,*j*)=0, otherwise. Each FCC lattice point has 12 neighbouring points and these 12 neighbours can be represented by 12 basis vectors: ***v***_1_=(1,1,0), ***v***_2_=(−1,−1,0), ***v***_3_=(−1,1,0), ***v***_4_=(1,−1,0), ***v***_5_=(0,1,1), ***v***_6_=(0,1,−1), ***v***_7_=(0,−1,1), ***v***_8_=(0,−1,−1), ***v***_9_=(1,0,1), ***v***_10_=(−1,0,1), ***v***_11_=(1,0,−1), ***v***_12_=(−1,0,−1). Each conformation can be encoded using these basis vectors. A sequence having *n* residues requires *n*−1 vectors to represent its conformation.

### 2.2 Contact-based energy model

In the literature, many researchers have proposed statistical energy models for protein structure prediction [7–9,35]. Empirical contact energies are calculated on the basis of information available from selected protein structures, with respect to a defined reference state, according to the quasi-chemical approximation. To each amino acid pair in contact, an additive energy is assigned and the energy of the protein can be estimated from the sum of all pairwise energies. In Berrera *et al.* [8], the authors derived contact energies from the potential of mean force theory relying on contact counts and solvent accessibility. They presented a table of empirical contact potential that gives the energy contributions associated to a pair of amino acids *s*_*a*_ and *s*_*b*_ in contact, described by the commutative function energy_BM_(*s*_*a*_,*s*_*b*_). These energy contributions are developed by applying statistical methods to the structures obtained from X-rays and NMR experiments. We will refer to this energy model as the BM energy model and also as the real energy function. The complete BM energy matrix is provided in the electronic supplementary material, table S1.

### 2.3 The problem model

Given the amino acid sequences *s*, a conformation *ϕ* of **s** in FCC lattice L is an injective function ϕ:1,…,n→L such that *ϕ*(*s*_*i*_) and *ϕ*(*s*_*i*+1_) are adjacent for *i*=1,…,*n*−1 and *ϕ*(*s*_*i*_)≠*ϕ*(*s*_*j*_) for *i*≠*j* to avoid overlapping. The protein folding problem can now be formalized as an optimization problem of finding the conformation *ϕ* of *s* such that the following energy function is minimized:

2.1 $$E(\phi )=\sum_{i=1}^{n-1}\sum_{j=i+2}^{n}\mathrm{contact}(\phi (s_{i}),\phi (s_{j}))\times {\mathrm{energy}}_{\mathrm{BM}}(s_{i},s_{j}).$$

### 2.4 Genetic algorithm

The genetic algorithm is a population-based framework which optimizes its population's fitness according to some well-defined optimization functions. The genetic algorithm improves its population by using a set of genetic operators on the current population. The operators produce children from the individuals of the current population. A selection strategy is then employed to select individuals from children and parents to form the next generation of population. The operators are traditionally divided into two classes: crossover and mutation. In a crossover operation, two individuals are selected from the population and fragments of their representations are interchanged to breed new individuals. Both the length and location of the fragments are randomly chosen. With a mutational operator, the representation of an individual is tweaked at random positions to get another valid individual.

In what follows, we provide the description of our algorithmic settings. In particular, we present the representation scheme, initialization, choice of genetic operators and their brief description, selection strategy and stagnation recovery technique that we have used in our approach. The strategy of deriving energy function by a non-uniform scaling is presented at the end of the section.

### 2.5 Representation

For the representation of a conformation we have used a scheme called *relative encoding* [36]. Relative encoding is implemented by choosing 12 different characters to represent 12 basis vectors for a FCC lattice unit. Our protein structure or conformation is represented by a string of these characters where each character represents a direction. Each direction vector in the string has a unique mapping in the three-dimensional coordinate system. If we follow the direction of the characters of the string consecutively, starting from the start of the string, we will have a walk in the protein chain with the conformation the string represents. A direction vector starts where its previous vector in the string ends at the three-dimensional coordinate. A string representing a valid conformation contains no loop, i.e. direction vectors do not form a cyclic path. Our population for the genetic algorithm is a set of these strings where each individual is an encoded structure. A conformation can easily be mapped into the three-dimensional space coordinate and vice versa.

### 2.6 Initialization

We have initialized the population for our genetic algorithm with random individuals following a simple random *chain growth initialization* method. Each individual is formed by choosing consecutive *n*−1 vectors. Each direction vector is chosen randomly from the 12 basis vectors of the FCC lattice unit. Notably, the formation of an individual as mentioned above has to be followed by a validity check so that the self-avoiding walk constraint is met.

### 2.7 Genetic operators

Our set of genetic operators includes crossover, pull move, diagonal move and rotation operator. The latter three operators are mutational operators. We have used these operators exhaustively, i.e. we apply an operator in all possible ways to an individual and select the best fit individual found during that process. Exhaustive use of operators has the penalty of an increased execution time. However, this is not a distinctive element of our algorithm. Many state-of-the-art algorithms including the works of Rashid *et al.* [12,37] have also used this scheme. It has been shown in the literature that exhaustiveness effectively reduces randomness in genetic algorithms [37]. The reduction of randomness directs the search towards a promising direction. Nevertheless, we have verified the effectiveness of exhaustive operator usage experimentally and the justification for its use is presented in the results section.

From some initial runs on smaller sequences we have observed that, after some generations, some operators have negligible or no contribution at all to the energy minimization, wasting a significant number of iterations of the algorithm. So instead of using a uniform probability distribution we have used a non-uniform distribution which is learned empirically from pilot runs. This is done to give bias to an operator which contributes largely to the energy minimization. At each generation, our algorithm chooses an operator randomly from this predefined probability distribution. Description of our chosen operators is given below:

(i) *Crossover*. Two parent conformations are selected from the population randomly and fragments of their string representations are interchanged at a selected point to generate two children. Two individuals survive from the children and the parents according to a selection strategy as described later.
(ii) *Pull move*. Pull moves were first proposed as a complete move set for lattices in the work of Lesh *et al.* [18] and later proved not to be completely reversible by Gyorffy *et al.* [38]. However, they are used extensively in the literature of protein folding simulation. An amino acid position in the three-dimensional space is selected as the pulling point. To this point, pulling can occur in both directions from the start of the amino acid chain or from the end of it. The current amino acid of the pulling point is moved to a neighbouring free point. And the rest of the amino acids are pulled in the direction of pulling to get a valid conformation.
(iii) *Diagonal move*. Diagonal moves are similar to the one monomer *kink jumps* used in the literature [39]. An amino acid position in the three-dimensional space is selected randomly. The selected amino acid is moved to a free neighbouring position. The move is only allowed when adjacent amino acids in the protein chain still remain adjacent after the move and no other constraints are violated.
(iv) *Rotation*. In a rotation move, starting from a random amino acid, the directions of the vectors in the current conformation are changed at random to find another valid conformation.

### 2.8 Selection strategy

We have used an elitist approach to select individuals for the next generation. Once an individual is generated by using an operator it survives in the next generation only if its parent is weaker in fitness than itself; otherwise we have kept the parent for the next generation.

### 2.9 Stagnation recovery

Our algorithm uses a traditional stagnation recovery technique called the *random walk* [40]. A number of successive pull moves are applied to randomly shuffle the individuals of the current population. This shuffling is done by control parameters to diversify the population structurally while keeping the average energy of the population lower.

### 2.10 Global algorithm

Our complete global algorithm is outlined in algorithm 1. Our algorithm starts with the initialization of the current population randomly. The algorithm terminates after a predefined time has elapsed and returns the best individual (BestGlobal) encountered during its complete execution. During the execution, the algorithm selects an operator randomly according to its probability distribution and applies the operator on the current population. After the application of the operator the new generation becomes the current population and its best individual (BestCurrent) is checked against the so far best individual (BestGlobal) for possible improvement and updating. A series of non-improving generation is tracked (trackingNonImprovement) and stagnation recovery is invoked after this tracking value crosses a predefined threshold (thresholdValue). During stagnation recovery, diversification of the current population is done by the use of the random walk technique. If two consecutive stagnation recoveries have no improvement between them then the shuffling parameter (shuffleParameter) is incremented which in turn causes more diversification in the later invocation.


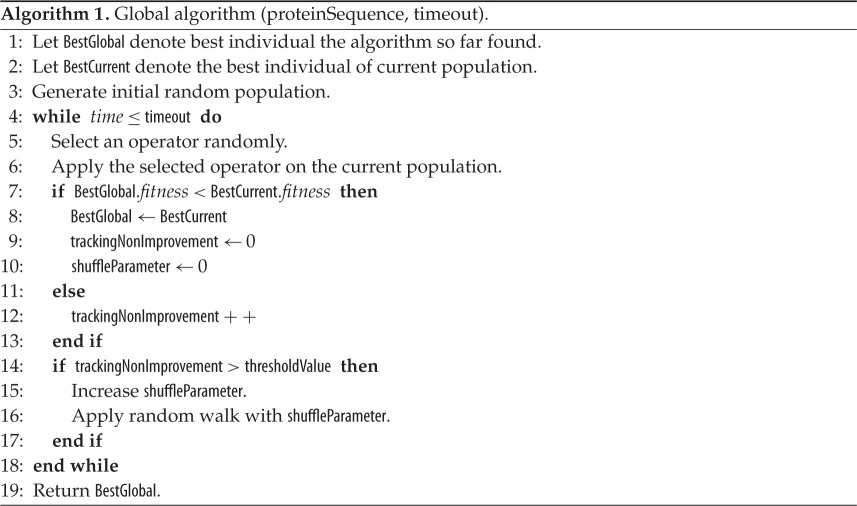


### 2.11 Non-uniform scaling of energy function

As has been stated earlier in the introductory section, the BM energy model [8] does not contain any information to distinguish among the contact types having different levels of magnitude of interaction potentials. In minimizing the energy function, contact types with negative energy contribution are desired. However, among these negative interactions, those with larger magnitude or absolute values are preferred over the interaction potentials with smaller magnitude or values. The search algorithm devoid of such information traps into local minima frequently. Initially, the contacts with interaction potential values larger in magnitude are preferred over the contacts with smaller energy magnitude. This helps to gain a lower energy structure very quickly. However, once a lower energy structure is formed, it is possible to establish the contacts with smaller energy magnitude that further lowers the energy level of the structure. We derive an energy function to achieve such properties within the energy model with a goal to guide the search more informatively.

At first, the entire BM energy levels are sorted with respect to their energy magnitudes. Subsequently, near energy levels are grouped together. Several groups are formed and each group is assigned with a group multiplier (*g*_*m*_). This multiplier is generated using the following analytical expression:

2.2 $$g_{m}=1+\frac{m\times (m+1)}{2},$$

here, *m* denotes the group number. All groups are sorted according to their energy levels and the numbers are assigned from 0 and onwards. A group gets higher *m* value if the energy magnitudes comprising the group are higher. Now, if a particular contact energy between two amino acid types *s*_*i*_ and *s*_*j*_ falls into group *m*, then the derived contact energy, energy_GW_(*s*_*i*_,*s*_*j*_) is defined by the following equation:

2.3 $${\mathrm{energy}}_{\mathrm{GW}}(s_{i},s_{j})=g_{m}\times {\mathrm{energy}}_{\mathrm{BM}}(s_{i},s_{j}),s_{i},s_{j}\in A.$$

For the ease of reference, we denote this formulation by group-weighted (GW) energy function in the rest of this paper. The analytical expression is chosen to achieve a nonlinear energy distribution from a nearly linear BM energy distribution. The aim of the above strategy is to keep the competition among contacts of a group active to some extent but to make the competition between the contacts of a low energy group and the contacts of a high energy group less prevalent.

From the formulation, it is evident that with a scaling factor, the GW energy function retains the information that is in the BM energy function and it also encapsulates the idea of making separation in different energy levels with a control of greediness. The control of greediness is in the choice of the number of groups. Choosing a very large number of groups will result in a very large group multiplier *g*_*m*_ for the group comprising energy levels of high magnitude. These large multipliers will create undesirable preferences for contacts falling in their respective groups. In the rest of this section, we present a qualitative description of the GW energy function with the help of an arbitrarily selected protein.

**Figure 1.**
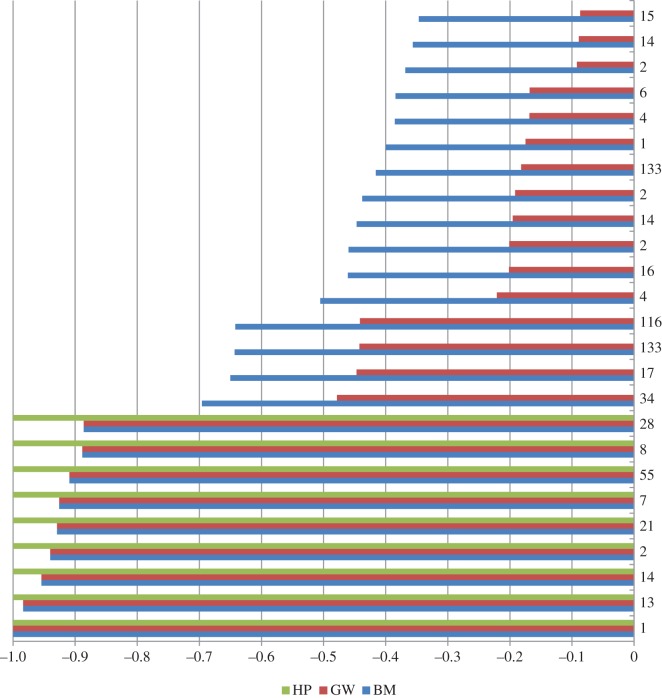
Comparison of partial energy distribution assigned by HP, GW and BM energy functions for protein 1CTF. Both GW and BM energy distributions are normalized.

Figure 1 depicts the partial energy distribution of various energy functions for the protein 1CTF of length 74 (full distribution is shown in the electronic supplementary material, figure S1). The BM and GW energy distribution are each normalized against their respective highest magnitude energy level. The number on the vertical axis in each row represents the number of potential contacts in 1CTF, each having the energy of the respective row value. Among 99 types of potential contacts present in 1CTF, first 25 contact energy levels in non-decreasing order are shown from bottom to top.

The protein 1CTF has 2628 potential contacts. Among them only 149 potential contacts are assigned energy value −1 in the HP energy distribution. These are shown as the sum of inscribed numbers in the lower nine green bars in figure 1. Almost 94% of the potential contacts are assigned 0 energy value. In figure 1, it is marked by the absence of green bars starting from the 10th bar from the bottom going upwards. There are a small number of potential contacts in BM energy function that are close to the contact energy level 0. So abruptly switching from −1 to 0 makes the case for HP being less informed. That is why HP energy function cannot contribute once the high energy contacts are formed. However, HP energy function was used as a guidance in Rashid *et al.* [12] along with the BM energy model.

In figure 1, it is also evident that GW retains all the relative differences of energy levels that BM contains with some scaling. Energy bars in red for GW energy function are smaller compared with blue bars for BM energy function. However, this signifies more pronounced separation between higher and lower energy levels in GW than in BM. Thus contacts between amino acid types in a higher group or level are preferred over those in the lower groups. Without these separations the search algorithm is less informed and would fall into local minima frequently, which is evident from the behavior of the search algorithm using BM energy function only.

HP energy function weighs only hydrophobic–hydrophobic (H−H) contribution which covers most of the higher energy levels in BM. This is why HP is so successful when used to drive the search initially. GW energy function captures this property of HP by assigning higher multiplicative factors for higher energy groups. In the process of making these high energy contacts' core as HP it now also can differentiate medium- and low-energy contacts to be facilitated into the core.

In GW energy function, the separation is achieved in absolute value, i.e. for both positive and negative energy levels. There are protein sequences containing significant number of contacts having high magnitude positive-energy levels and GW can contribute from both sides for these types of proteins because it favours high magnitude negative-energy contacts to be formed and at the same time high magnitude positive energy contacts to be inhibited.

## 3. Results and discussion

We have implemented our algorithms in Java programming language and have conducted our experiments using several local computers. Each computer was equipped with an Intel Core i5-3470 processor @3.2 GHz clock speed having 6M smart cache and 4 GB of memory. The operating system installed was Ubuntu 13.04 OS. The code is freely available at: https://github.com/swakkhar/ProteinFoldingGW.

### 3.1 Benchmark proteins and settings of parameters

Our selected benchmark proteins are shown in table 1. The first seven protein sequences are taken from the Protein Data Bank (PDB) database and were used in the work of Ullah *et al.* [11]. The middle five sequences are taken from CASP9 and were first used by Shatabda *et al.* [13]. These proteins are of length ranging from 54 to 160. All these benchmark proteins were previously used by Rashid *et al.* [12] and Shatabda *et al.* [13]. To the best of our knowledge, the last five sequences have been used in the literature only for the HP model. As a result, we are unable to make a fair comparison with any other model from the literature for these five sequences. We have only presented results of these sequences to demonstrate the effectiveness of our model on larger sequences.

**Table 1.** Real benchmark sequences selected for experiment from PDB and CASP9.

PDB ID	length	sequence
4RXN	54	MKKYTCTVCGYIYNPEDGDPDNGVNPGTDFKDIPDDWVCPLCGVGKDQFEEVEE
1ENH	54	RPRTAFSSEQLARLKREFNENRYLTERRRQQLSSELGLNEAQIKIWFQNKRAKI
4PTI	58	RPDFCLEPPYTGPCKARIIRYFYNAKAGLCQTFVYGGCRAKRNNFKSAEDCMRTCGGA
2IGD	61	MTPAVTTYKLVINGKTLKGETTTKAVDAETAEKAFKQYANDNGVDGVWTYDDATKTFTVTE
1YPA	64	MKTEWPELVGKAVAAAKKVILQDKPEAQIIVLPVGTIVTMEYRIDRVRLFVDKLDNIAQVPRVG
1R69	69	SISSRVKSKRIQLGLNQAELAQKVGTTQQSIEQLENGKTKRPRFLPELASALGVSVDWLLNGTSDSNVR
1CTF	74	AAEEKTEFDVILKAAGANKVAVIKAVRGATGLGLKEAKDLVESAPAALKEGVSKDDAEALKKALEEAGAEVEVK
3MX7	90	MTDLVAVWDVALSDGVHKIEFEHGTTSGKRVVYVDGKEEIRKEWMFKLVGKETFYVGAAKTKATINIDAISGFAY
		EYTLEINGKSLKKYM
3NBM	108	SNASKELKVLVLCAGSGTSAQLANAINEGANLTEVRVIANSGAYGAHYDIMGVYDLIILAPQVRSYYREMKVDA
		ERLGIQIVATRGMEYIHLTKSPSKALQFVLEHYQ
3MQO	120	PAIDYKTAFHLAPIGLVLSRDRVIEDCNDELAAIFRCARADLIGRSFEVLYPSSDEFERIGERISPVMIAHGSYA
		DDRIMKRAGGELFWCHVTGRALDRTAPLAAGVWTFEDLSATRRVA
3MR0	142	SNALSASEERFQLAVSGASAGLWDWNPKTGAMYLSPHFKKIMGYEDHELPDEITGHRESIHPDDRARVLAALKAHL
		EHRDTYDVEYRVRTRSGDFRWIQSRGQALWNSAGEPYRMVGWIMDVTDRKRDEDALRVSREELRRL
3PNX	160	GMENKKMNLLLFSGDYDKALASLIIANAAREMEIEVTIFCAFWGLLLLRDPEKASQEDKSLYEQAFSSLTPREAEEL
		PLSKMNLGGIGKKMLLEMMKEEKAPKLSDLLSGARKKEVKFYACQLSVEIMGFKKEELFPEVQIMDVKEYL
		KNALESDLQLFI
3MSE	179	ISPNVLNNMKSYMKHSNIRNIIINIMAHELSVINNHIKYINELFYKLDTNHNGSLSHREIYTVLASVGIKKWDINR
		ILQALDINDRGNITYTEFMAGCYRWKNIESTFLKAAFNKIDKDEDGYISKSDIVSLVHDKVLDNNDIDNFFLSVHS
		IKKGIPREHIINKISFQEFKDYMLSTF
3MR7	189	SNAERRLCAILAADMAGYSRLMERNETDVLNRQKLYRRELIDPAIAQAGGQIVKTTGDGMLARFDTAQAALRCALE
		IQQAMQQREEDTPRKERIQYRIGINIGDIVLEDGDIFGDAVNVAARLEAISEPGAICVSDIVHQITQDRVSEPFTD
		LGLQKVKNITRPIRVWQWVPDADRDQSHDPQPSHVQH
3NO6	229	MTFSKELREASRPIIDDIYNDGFIQDLLAGKLSNQAVRQYLRADASYLKEFTNIYAMLIPKMSSMEDVKFLVEQIEFML
		EGEVEAHEVLADFINEPYEEIVKEKVWPPSGDHYIKHMYFNAFARENAAFTIAAMAPCPYVYAVIGKRAMEDPKLN
		KESVTSKWFQFYSTEMDELVDVFDQLMDRLTKHCSETEKKEIKENFLQSTIHERHFFNMAYINEKWEYGGNNNE
3NO3	258	MNLKSTLLLLLCLMMAGMVAAKDNTKVIAHRGYWKTEGSAQNSIRSLERASEIGAYGSEFDVHLTADNVLVVYHD
		NDIQGKHIQSCTYDELKDLQLSNGEKLPTLEQYLKRAKKLKNIRLIFELKSHDTPERNRDAARLSVQMVKRMKLA
		KRTDYISFNMDACKEFIRLCPKSEVSYLNGELSPMELKELGFTGLDYHYKVLQSHPDWVKDCKVLGMTSNVWTV
		DDPKLMEEMIDMGVDFITTDLPEETQKILHSRAQ
3ON7	279	MKLETIDYRAADSAKRFVESLRETGFGVLSNHPIDKELVERIYTEWQAFFNSEAKNEFMFNRETHDGFFPASISE
		TAKGHTVKDIKEYYHVYPWGRIPDSLRANILAYYEKANTLASELLEWIETYSPDEIKAKFSIPLPEMIANSHKT
		LLRILHYPPMTGDEEMGAIRAAAHEDINLITVLPTANEPGLQVKAKDGSWLDVPSDFGNIIINIGDMLQEASD
		GYFPSTSHRVINPEGTDKTKSRISLPLFLHPHPSVVLSERYTADSYLMERLRELGVL

In the implementation of our genetic algorithms we have used a population size of 50. The threshold values used for invoking the random walk are summarized in table 2. As has been mentioned in the methods section, at each generation, our algorithm chooses an operator randomly from a predefined probability distribution learned empirically from pilot runs. During the pilot runs we have observed that, after some iterations, some operators contribute less to the energy minimization than others. This non-uniform probability distribution is used to give bias to an operator which contributes more to the energy minimization. The selection probability for pull move, crossover, corner flip and rotation operators are 0.40, 0.25, 0.20 and 0.15, respectively. All of these parameters are crudely optimized from several pilot runs.

**Table 2.** Threshold values for invoking the random walk in the global algorithm.

PDB ID	length	threshold value
4RXN	54	50
1ENH	54	50
4PTI	58	50
2IGD	61	50
1YPA	64	45
1R69	69	45
1CTF	74	40
3MX7	90	35
3NBM	108	30
3MQO	120	25
3MR0	142	20
3PNX	160	15
3MSE	179	15
3MR7	189	15
3NO6	229	15
3NO3	258	15
3ON7	279	15

### 3.2 Settings for group-weighted energy function

Our genetic algorithm was driven by the GW energy function. However, we only report the BM energy function values returned by our algorithm. In order to derive the GW energy function levels, we divided the entire BM energy levels into six groups and used a scaling factor as described in the previous section. Settings of our GW energy function are summarized in table 3. In particular, table 3 reports the multiplier value derived using equation (2.2) along with the corresponding range of the magnitude of the contact types for each group. Note that using a single group in the GW energy function setting is equivalent to the direct use of BM energy function. We have settled for six groups based on some preliminary experiments. Our choice of group settings demonstrates the usefulness of non-uniform scaling strategy. (Comparative differences with the real energy function under this setting are presented in the electronic supplementary material, figure S1. The differences are sequence independent but figure S1 they are presented considering protein 1CTF to make it visibly concrete in terms of the number of contacts.)

**Table 3.** Settings for the GW energy function: |energy_BM_| denotes the magnitude of the energy level using the BM energy level.

group (*m*)	*g*_*m*_	|energy_BM_|×10^3^
5	16	2000−3477
4	11	1501−2000
3	7	1001−1500
2	4	501−1000
1	2	101−500
0	1	0−100

### 3.3 Comparison with the state-of-the-art results

We have conducted 50 independent runs for each of the protein sequences. Each run is allowed a time-span of 2 h. In table 4, we show the comparison between our results and the state-of-the-art results achieved by Rashid *et al.* [12]. Rahisd *et al.* [12] used a mixed energy model to drive the genetic algorithm and enforced a 1-h cut-off time for each of the protein sequences. Best and average energy levels for each of the proteins found by their algorithm are reported under the column titled ‘mixed model’. For a fair comparison, we also report the best and average energy levels found from our 50 independent runs by taking the values after 1 h only. The results from our algorithm are reported in the column titled ‘GW function’. The better results are highlighted using boldfaced fonts. Note that we are minimizing the energy function and lower values are better. Our algorithm is able to produce better average and best energy levels for all the 12 proteins. Moreover, we report the relative improvement (R.I.) achieved by our algorithm for the best and average energy levels with respect to the algorithm in [12]. R.I. is defined as follows:

3.1 $$\mathrm{R.I.}=\frac{E_{t}-E_{r}}{E_{r}}\times 100\%$$

**Table 4.** The energy values achieved by our algorithm using the derived energy function or GW function and the genetic algorithm of Rashid *et al.* [12] using the mixed energy model. Bold indicates the better values.

		mixed model [12]		GW function		R.I. (%)	
PDB ID	length	best	avg	best	avg	best	avg
4RXN	54	−166.88	−162.72	−**170.27**	−**167.71**	2.03	3.06
1ENH	54	−153.79	−151.65	−**158.5**	−**155.13**	3.06	2.29
4PTI	58	−210.29	−204.56	−**215.8**	−**210.92**	2.62	3.11
2IGD	61	−183.18	−176.83	−**188.62**	−**185.04**	2.96	4.64
1YPA	64	−256.95	−253.09	−**261.54**	−**257.42**	1.78	1.71
1R69	69	−216.37	−208.79	−**223.91**	−**217.86**	3.48	4.34
1CTF	74	−233.51	−225.43	−**239.22**	−**233.58**	2.44	3.61
3MX7	90	−340.05	−325.45	−**348.69**	−**341.14**	2.54	4.82
3NBM	108	−436.76	−419.25	−**454.17**	−**441.01**	3.98	5.19
3MQO	120	−486.05	−472.78	−**507.17**	−**486.55**	4.34	2.91
3MRO	142	−479.36	−447.77	−**491.1**	−**468.99**	2.44	4.73
3PNX	160	−615.82	−592.25	−**639.62**	−**610.38**	3.86	3.06
3MSE	179	—	—	−**744.41**	−**703.85**	—	—
3MR7	189	—	—	−**683.61**	−**650.01**	—	—
3NO6	229	—	—	−**858.13**	−**820.9**	—	—
3NO3	258	—	—	−**940.28**	−**895.88**	—	—
3ON7	279	—	—	−**991.91**	−**958**	—	—

Here, for each protein, the relative improvement of the target (t) with respect to the reference (r) is calculated using equation (3.1), where *E*_t_ and *E*_r_ denote the average or best energy values achieved by target and reference, respectively. R.I. for both best and average energy levels are reported in the column ‘R.I’ in table 4. This metric was also used by Rashid *et al.* [12] to denote the significance in improvement in the energy level. Generally, it is harder to improve the values as they get closer to lower energy values. Relative improvement normalizes the improvement value by taking the percentage over the reference value. The values reported in the table clearly show the significance of our improvement.

**Figure 2.**
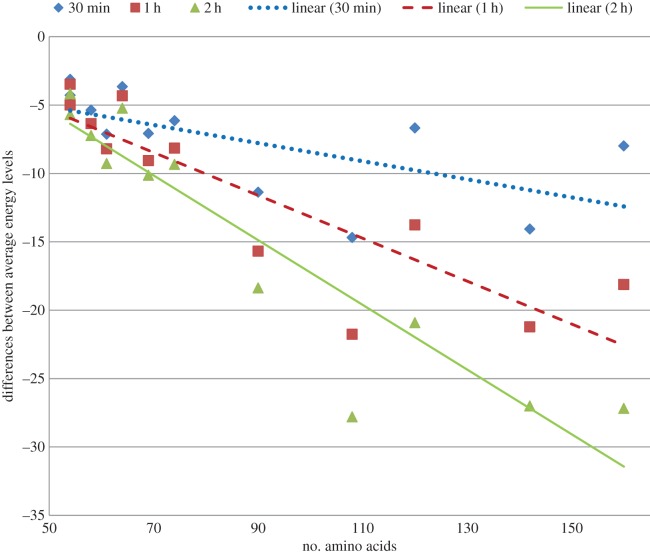
Differences of average energy obtained by using derived energy function and mixed model [12].

**Figure 3.**
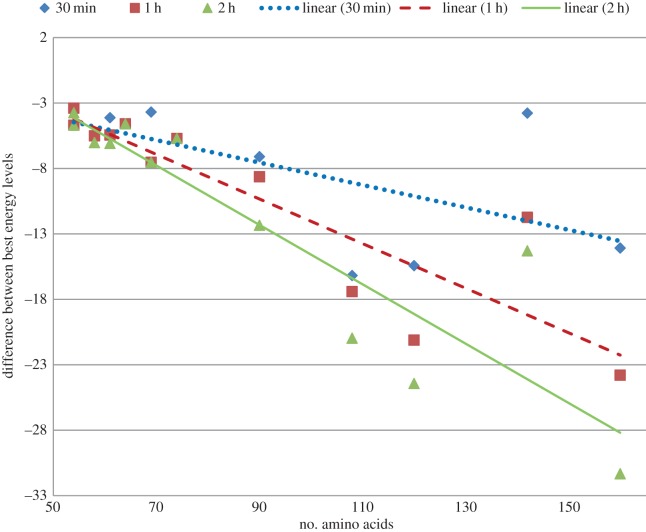
Differences of best energy obtained by using derived energy function and mixed model [12].

**Figure 4.**
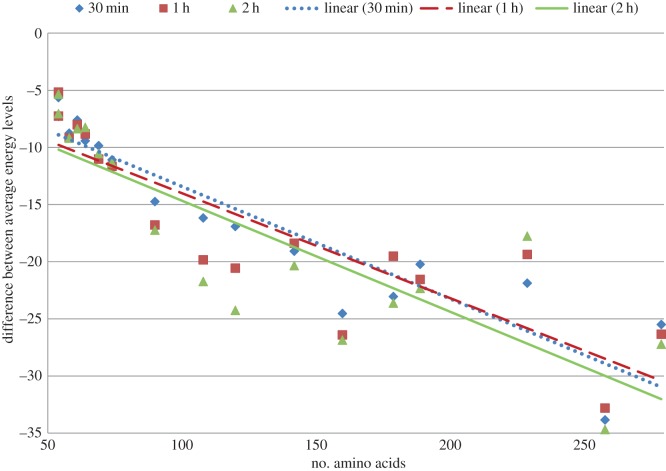
Differences of average energy obtained by using derived energy function (GW) and using real energy function (BM).

**Figure 5.**
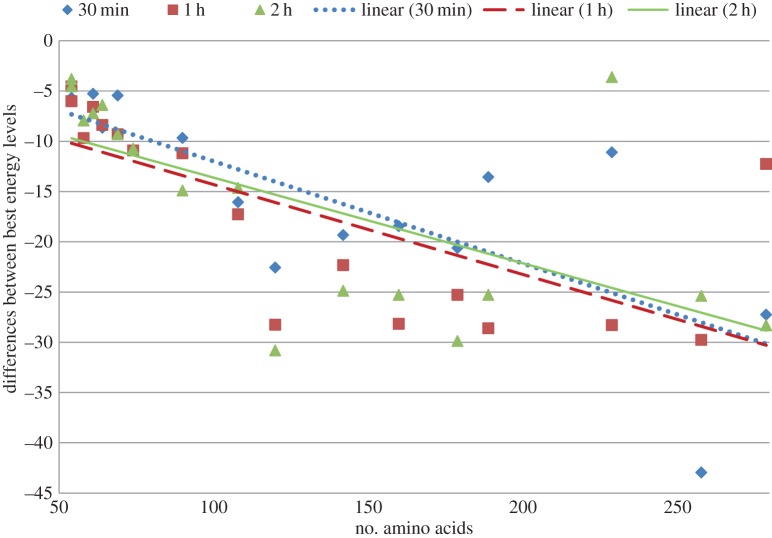
Differences of best energy obtained by using derived energy function (GW) and using real energy function (BM).

**Figure 6.**
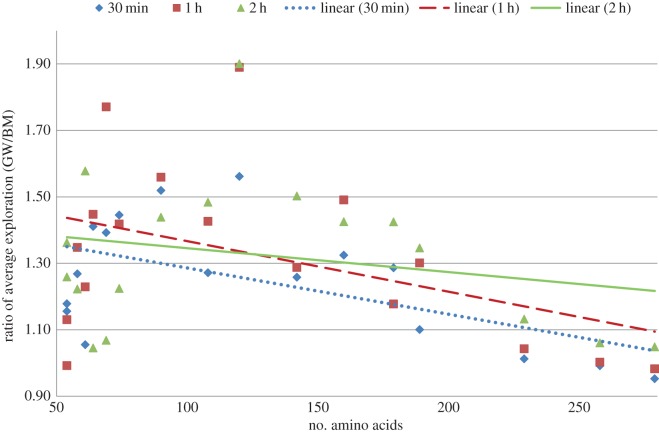
Ratio of average number of objective function evaluation by using derived energy function (GW) to the same using real energy function (BM).

**Figure 7.**
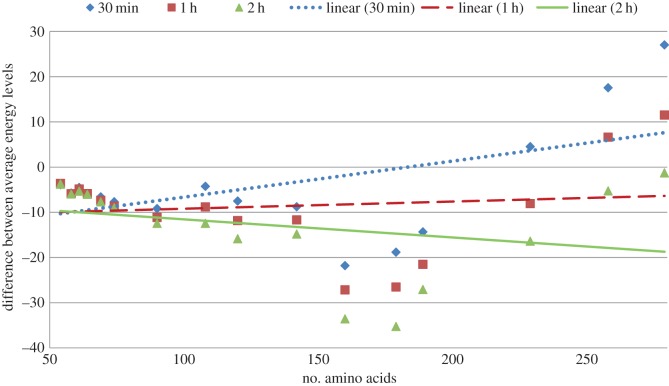
Differences of average energy obtained by using derived energy function exhaustively (GW) and using derived energy function non-exhaustively (GWN).

Furthermore, we also report the improvement of our approach over the algorithm of Rashid *et al.* [12] based on the the average and best energy levels recorded after 30 min, 1 h and 2 h in figure 2 and in figure 3, respectively. In these figures we plot the improvement achieved by our algorithms for different run times for each of the proteins. We clearly see that even our 30 min energy values are lower than the values achieved by Rashid *et al.* [12]. We also show our energy levels at the 2 h cut-off time to illustrate that our approach can achieve further improvement if the cut-off time is delayed. Three distinct linear trend lines with downward slope suggest that with the increase in dimensionality or size of the protein sequence our approach performs better. Exact energy values used to generate figures 2 and 3 are presented in the electronic supplementary material, table S2. Informatively, in figures 2–7, each of the linear trend lines is the result of the linear regression of the dataset of each time interval as follows. Each line is based on the equation: *y*=*mx*+*c*, where $m=\left(n\sum (xy)-\sum (x)\sum (y)\right)/\left(n\sum (x^{2})-{\left(\sum (x)\right)}^{2}\right)$ and c=(∑(y)−m∑(x))/n. Here *n* is number of data points, *x*'s are the number of amino acids and *y*'s are the differences of energies or ratios of the exploration metric.

We have also compared the best energy values achieved with our GW function and the results reported by Ullah *et al.* in [11]. Ullah *et al.* [11] achieved state-of-the-art results in terms of best energy values using a hybrid algorithmic approach incorporating large neighbourhood search and constraint programming. In table 5, we present our best energy values of 50 independent runs and the corresponding time to reach the best energy level. Clearly, in all the seven benchmark proteins, our energy values are found to be significantly lower. Notably, some of the best energy values are found within a very short time. However, for the larger protein sequences their algorithm could not produce any valid conformation even after running it for a long time [13].

**Table 5.** The best energy values achieved by our approach using the GW function and the hybrid approach [11]. Bold indicates the better values.

		hybrid approach [11]		our GW function	
pdb id	length	energy	time	energy	time
4RXN	54	−168.076	1 h 5 min	−**170.618**	1 h 56 min
1ENH	54	−157.062	1 h 2 min	−**158.504**	21 min
4PTI	58	−213.778	1 h 20 min	−**216.311**	1 h 54 min
2IGD	61	−186.696	55 min	−**189.27**	1 h 28 min
1YPA	64	−258.709	42 min	−**261.535**	18 min
1R69	69	−222.317	35 min	−**223.914**	58 min
1CTF	74	−233.764	1 h 36 min	−**239.218**	13 min

### 3.4 Comparison with the BM energy function

To illustrate the effectiveness of our GW energy function we run the same set of experiments using the real energy function (BM) to guide the genetic algorithm. Throughout the experiments, the same algorithmic settings are used. The difference is only in the search guidance. Figures 4 and 5 illustrate the differences in average and best energy levels achieved by the same genetic algorithm using different guidance. The improvement achieved by the genetic algorithm using GW energy function is shown for each of the proteins for different cut-off times. As is evident from the figures, the differences of both best and average values plotted against the protein lengths form downward sloping trend lines. These differences clearly denote the superiority of the GW energy function over the BM energy function in search guidance. Also, the differences become more pronounced when the dimensionality or the size of the protein sequence increases. This is also an indication of the scalability of our approach. The exact values to generate these figures and average, best, median, standard deviation of the energy levels for both GW-guided and BM-guided algorithms are presented in the electronic supplementary material, table S3.

### 3.5 Similarity with native structures

In order to assess the quality of generated structures, we report the root mean square distance (RMSD) measure with native structures already determined using *in vitro* laboratory methods. RMSD measure is calculated according to the following equation:

3.2 $$\mathrm{RMSD}(\phi_{o},\phi_{n})=\sqrt{\frac{\sum_{i=1}^{n-1}\sum_{j=i+1}^{n}{\left(d_{ij}(\phi_{o})-d_{ij}(\phi_{n})\right)}^{2}}{n\times (n-1)/2}},$$

here, *ϕ*_*o*_ is the observed or generated structure by our method and *ϕ*_*n*_ is the native structure stored in the PDB. We read only the *α*-carbon coordinates from the PDB file as our model represents each amino acid by a single point in the lattice. For two corresponding amino acids in protein sequences *i* and *j*, *d*_*ij*_(*ϕ*_*o*_) denotes the Euclidean distance between the points they are mapped to in the conformation *ϕ*_*o*_. Best and average RMSD measures in angstroms are reported in table 6 for different settings of our algorithm and for the mixed energy model in Rashid *et al.* [12]. The first two columns under the common heading ‘initial rmsd’ show the quality of the structures at the beginning of runs by our algorithm. The next two columns under the common heading ‘best conformation’ correspond to the RMSD of the best conformation found by minimizing the energy function. We note that there is not much improvement in the RMSD values of the best structures from the initial random structures. This indicates the absence of strong positive correlation between the value of the energy function and the RMSD measure. Similar findings are also reported in Shatabda *et al.* [34]. Details of the energy function values and corresponding RMSD values are given in the electronic supplementary materials, table S4. Furthermore, even if we take the top 2000 best conformations according to the minimized energy function the situation remains almost unchanged. Best and average RMSD of such an ensemble of the top 2000 structures are reported in the next two columns.

**Table 6.** Best and average RMSD values achieved by different ensembles of our algorithm and by the mixed energy model [12]. Bold indicates the better values.

		initial RMSD		best conformation		best 2000 conformation		all conformation		mixed model [12]	
PDB ID	length	avg	best	avg	best	avg	best	avg	best	avg	best
4RXN	54	6.2	5.07	6.76	6.24	6.15	5.9	**4.34**	**4.03**	5.41	4.7
1ENH	54	6.29	4.57	6.91	6.51	6.33	6.07	**4.07**	**3.77**	5.22	4.57
4PTI	58	7.51	6.32	7.99	7.67	7.44	6.98	**5.13**	**4.65**	6.46	5.97
2IGD	61	8.77	7.45	9.32	8.98	8.86	8.41	**5.73**	**5.32**	7.81	6.85
1YPA	64	7.57	6.29	7.67	7.25	7.19	6.85	**5.05**	**4.73**	6.29	5.42
1R69	69	6.66	4.9	6.55	6.15	6.01	5.66	**4.03**	**3.7**	5.17	4.68
1CTF	74	7.91	6.17	7.13	6.57	6.68	6.18	**5.7**	**4.99**	5.28	4.69
3MX7	90	9.32	7.92	9.5	9.08	9.12	8.74	**7.1**	**6.78**	7.94	7.31
3NBM	108	9.32	7.41	8.56	7.98	8.2	7.57	6.66	5.91	**6.46**	**5.58**
3MQO	120	9.63	7.75	9.45	8.73	9.09	8.51	7.06	6.62	**6.84**	**6.17**
3MRO	142	11.86	9.28	13.06	11.88	12.75	11.52	8.81	8.01	**8.72**	**7.65**
3PNX	160	12.06	9.2	11.59	10.5	11.34	10.22	**8.36**	**7.26**	8.51	7.5
3MSE	179	16.85	11.21	19.7	17.89	18.83	15.26	9.29	7.7	—	—
3MR7	189	12.67	9.48	10.88	9.98	10.58	9.74	8.83	7.95	—	—
3NO6	229	14.34	10.78	12.76	11.84	12.32	11.38	10.29	9.37	—	—
3NO3	258	14.53	9.84	11.07	9.68	9.82	8.78	8.56	7.86	—	—
3ON7	279	15.4	11.76	13.27	12.26	11.99	10.92	11.02	10.41	—	—

We also report the best and average RMSD values reported by Rashid *et al.* [12] in the last two columns of table 6. In Rashid *et al.* [12] reported the best and average RMSD values for the best RMSD value found among all conformations in the entire run. For a fair comparison, we also report the same for our algorithm in the columns under the heading ‘all conformation’. From the reported values we could note that for nine benchmark protein sequences out of 12, our method yields structures with lower RMSD values. Better values are shown in boldfaced fonts in the table. One obvious reason behind this significant improvement is that at the initial stage of energy optimization a hydrophobic core is formed in the centre by the method in Rashid *et al.* [12] which discards any other type of interactions except H−H. Moreover, they deploy a hydrophobic core based macro-mutation operator that repetitively converges to a core formed by the HP energy model which is not able to distinguish between other types of contacts. In our approach, we are able to distinguish also the other types of interactions. As a result, after the initial formation of the hydrophobic core, the structure is further refined by other types of interactions which also resemble the physical folding phenomena.

### 3.6 Comparison of efficiency

To evaluate the performance of an algorithm, number of objective function evaluation during a single run of the algorithm as a comparison metric has been used in the literature. This provides us with a comparison metric that is independent of the underlying hardware and implementation. Previous works that used this metric for comparison are mostly based on HP and MJ energy models. We have found only one work that is equivalent to our model. Maher *et al.* [26] have used FCC lattice and BM energy model in their work and have provided objective function evaluation data for four small sequences. In table 7, we have compared the work of Maher *et al.* [26] with our algorithm guided by GW function. In table 7, *E*_avg_ is the average energy value and Eval_*yyy*_'s are the numbers of objective function evaluation of Maher *et al.* [26] where *yyy*∈{min, avg, max}. To make a valid comparison we have provided energy value that is slightly lower (i.e. better) than Maher *et al.* [26], denoted by *Z*_avg_. The corresponding numbers of objective function evaluation are denoted by Oval_*yyy*_'s (*yyy*∈{min, avg, max}). Speed-up is defined as follows:

3.3 $$\mathrm{Speed-up}=\frac{{\mathrm{Eval}}_{\mathrm{avg}}}{{\mathrm{Oval}}_{\mathrm{avg}}}$$

**Table 7.** Comparison of the number of objective function evaluation achieved by our algorithm using the derived energy function or GW function to the same achieved by the firefly inspired algorithm in Maher *et al.* [26].

		firefly algorithm [26]				GW function				
PDB ID	length	E_avg_	${\mathrm{Eval}}_{\min}$ ×10^6^	Eval_avg_ ×10^6^	${\mathrm{Eval}}_{\max}$ ×10^6^	Z_avg_	${\mathrm{Oval}}_{\min}$ ×10^6^	Oval_avg_ ×10^6^	${\mathrm{Oval}}_{\max}$ ×10^6^	speed-up
4RXN	54	−158.9	20.05	39.82	52.78	−159.32	0.44	1.86	6.22	21.41
1ENH	54	−144.12	16.86	35.09	47.52	−144.48	0.18	0.81	1.88	43.32
4PTI	58	−200.86	23.98	31.88	58.56	−201.32	0.48	2.24	8.27	14.23
2IGD	61	−179.88	34.71	64.87	111.22	−180.28	1.01	7.03	24.13	9.23
average speed-up										22.05

In table 7, we see that average speed-up is more than 22 times which indicates that the energy values provided in Maher *et al.* [26] are surpassed by our algorithm within a few iterations at a very early stage. Since state-of-the-art works lack results on larger sequences for this comparison metric, we have provided a comparison of the numbers of energy function evaluation between GW function and BM energy function in table 8. In table 8, the average energy values of BM function and corresponding numbers of energy function evaluation after a 2 h runtime have been reported. To make a valid comparison we have provided energy values of GW function that are slightly lower (i.e. better) than the respective energy values of the BM energy function. We observe more than seven times average speed-up in this comparison.

**Table 8.** Comparison of the number of objective function evaluation achieved using the derived energy function or GW function to the same achieved using the real energy function (BM).

		BM function				GW function				
PDB ID	length	E_avg_	${\mathrm{Oval}}_{\min}$ ×10^6^	Oval_avg_ ×10^6^	${\mathrm{Oval}}_{\max}$ ×10^6^	Z_avg_	${\mathrm{Oval}}_{\min}$ ×10^6^	Oval_avg_ ×10^6^	${\mathrm{Oval}}_{\max}$ ×10^6^	speed-up
4RXN	54	−161.35	1	71.61	179.69	−161.78	0.48	3.37	12.4	21.25
1ENH	54	−150.46	2.51	64.23	181.46	−150.78	0.68	4.42	15.28	14.53
4PTI	58	−202.58	1.57	56.07	160.15	−203.03	0.6	3.48	12.87	16.11
2IGD	61	−177.73	4.2	52.13	153.69	−178.17	0.54	4.41	19.71	11.82
1YPA	64	−250.06	4.11	67.99	143.5	−250.5	0.65	5.19	12.95	13.10
1R69	69	−208.37	2.12	53.51	125.68	−208.83	0.97	5.77	18.2	9.27
1CTF	74	−223.41	1.01	53.37	118.47	−223.92	0.67	6.68	31.96	7.99
3MX7	90	−326.57	2.26	39.68	93.62	−327.14	1.11	6.99	59.23	5.68
3NBM	108	−425.3	2.1	34.41	69.77	−425.78	1.3	11.11	79.4	3.10
3MQO	120	−469.43	2.11	25.48	61.73	−469.92	1.12	10.23	61.01	2.49
3MRO	142	−454.42	3.58	27.02	49.41	−454.59	1.78	10.34	57.98	2.61
3PNX	160	−592.57	3.41	23.47	40.1	−593.22	1.72	8.19	27.2	2.87
3MSE	179	−692.76	3.58	22	34.86	−693.34	2.03	12.55	50.5	1.75
3MR7	189	−636.64	3.83	20.17	32.61	−635.79	1.44	11.82	57.59	1.71
3NO6	229	−817.31	5.63	19.39	24.58	−816.23	2.88	12.76	32.18	1.52
3NO3	258	−880.46	0.84	17.37	21.2	−881.13	1.89	7.98	24.98	2.18
3ON7	279	−953.87	5.38	16.14	18.63	−954.63	2.43	8.76	21.48	1.84
average speed-up										7.05

It seems evident from table 8 that the speed-up value decreases with the increase of the length of the protein sequences. From figure 6, we observe that initially for the largest two sequences the ratio of average exploration of GW to that of BM is less than 1. However, subsequently this ratio increases gradually, and after the 2 h cut-off time all the ratios are greater than 1. The trend line in figure 6 flattens as time progresses. This is because hitting local minima frequently requires more time for the larger sequences as compared with the smaller ones and as soon as the algorithm starts hitting local minima frequently the BM-guided algorithm starts lagging in comparison to the GW-guided algorithm. This indicates that if the time limit is further extended for the larger sequences (e.g. 4 h instead of 2 h) then we can expect to get large speed-up values for the longer sequences. Exact values to generate figure 6 as well as corresponding energy values of each time interval are provided in the electronic supplementary material, table S5.

### 3.7 Effectiveness of exhaustive operator usage

As has been mentioned in the methods section, we have experimentally verified the effectiveness of the exhaustive use of the genetic operators. For this purpose, we have run two versions of our algorithm using the same GW energy function. One version uses operators exhaustively; we denote this version as GW. The other version runs the global algorithm as before but during the application of an operator does not apply the operator exhaustively; we denote this latter version as GWN. Figure 7 shows the differences between the average energy values of GW and that of GWN. From the figure it is evident that initially GWN poses superiority where the protein sequences are larger, but as the time progresses GW starts to dominate. In the smaller sequences, GW dominates from the start of the algorithm. This is because for a larger sequence the algorithm requires longer time to hit the local minima; once the algorithm starts to hit local minima frequently, exhaustive generation starts to scale well.

**Figure 8.**
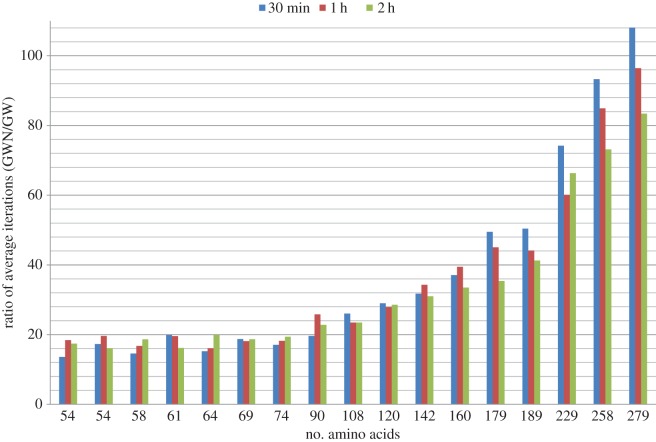
Ratio of average number of iterations by using derived energy function non-exhaustively (GWN) to the same using derived energy function exhaustively (GW).

In figure 8, we see the ratio of average number of iterations of GW to that of GWN. For smaller sequences the ratios are within the same range. As the sequence gets larger the ratio increases abruptly. This is because the conformation of a larger sequence gets perturbed more by an operator to generate different conformations. So in a single step of the algorithm, the exhaustive version requires more time but this results in a lesser number of global iterations. In summary, the non-exhaustive version iterates more globally but lags in terms of energy values. Exact values to generate figures 7 and 8 as well as the average, best, median and standard deviation of energy values for both the algorithms are provided in the electronic supplementary material, table S6.

## 4. Conclusion and future work

Efficient conformational space sampling is a long standing issue in the domain of *ab initio* protein folding simulation. In this paper, we have presented a new way to tackle this sampling issue. The energy functions at hand are not very informative for a search algorithm to find a low-energy conformation of a protein. We have proposed a strategy in which a non-uniformly scaled version of an energy function is used to guide the search algorithm. This derived energy function after scaling remains directly correlated with the real energy function. The derived energy function is integrated with favourable properties that make it suitable for efficient searching. This informed energy function has helped our search algorithm to explore such regions of the conformational space that no previous methods have been able to explore. In all the benchmark proteins, the derived energy function has improved the state-of-the-art results but the improvement is much more pronounced when the size of the protein sequence increases. In the comparison of efficiency metric, our method has shown unparalleled improvement over the-state-of-art algorithms.

Our method has also been proved to be effective in generating structures with increased similarity with the native structures when compared with the previous best algorithms. Even though the RMSD measures to native structures do not show much improvement, this negative aspect is not tied to our method; rather it is tied to the statistical energy models we have used [8] in our algorithm. Our algorithm successfully samples lower energy conformations; in theory these are supposed also to show lower RMSD measures to native structures. But here we observe an absence of strong correlation of RMSD measures and the conformational energy. This can be attributed to the limitations of model assumption in designing a statistical- or knowledge-based energy model. Currently available knowledge-based energy functions show poor performance when they are tested on sequences outside of their knowledge domain. The poor correlation of conformational energy and RMSD measure is another hurdle that remains to be overcome in the domain of *ab initio* modelling. Our approach is loosely coupled with the underlying statistical energy model. If we replace the underlying energy model with a better model our method will still be relevant for the efficient exploration of conformational search space.

The strategy we used here to derive energy function is simple and domain independent. Most of the optimization problems require a heuristic function that is well equipped with a direction for efficient search and information for better optimization. Our strategy thus can also be extended to those real-life optimization problems for improvement. The technique of deriving the energy function can be readily adapted and extended to fuse other information in the function. For such a fusion, an instant candidate that comes from the observation of partial energy distribution in figure 1 is the number of potential contacts of an energy level. If a protein contains a certain type of potential contacts more than the other types of contacts, more weight can be put on this certain type of potential contact. It will then increase the likelihood of the formation of this certain type of contacts in the population. However, giving weight to a type of contacts should capture their energy levels; otherwise search algorithms will form contacts that are frequent in a protein sequence but less in energy contribution. Therefore, the potential number of contacts of an energy level and magnitude of energy level information can be integrated in a single function with a suitable weighting scheme. We believe that this hybrid function is an interesting topic for exploration and further research in the domain of *ab initio* protein folding simulation.

## Supplementary Material

Table S5.xlsx

## Supplementary Material

Table S6.xlsx

## Supplementary Material

Figure S1.xlsx

## Supplementary Material

Table S1.xlsx

## Supplementary Material

Table S2.xlsx

## Supplementary Material

Table S3.xlsx

## Supplementary Material

Table S4.xlsx

## Data accessibility

The Real benchmark sequences used in our experiments are reported in table 1. Detailed data of our experiments are available as the electronic supplementary material files.
